# Cesarean Delivery of a Dichorionic Diamniotic Twin Pregnancy Complicated With a Complete Septate Uterus: A Case of Septum Perforation to Deliver the Trailing Twin

**DOI:** 10.7759/cureus.78523

**Published:** 2025-02-04

**Authors:** Emi Nakamoto, Takuya Misugi, Kohei Kitada, Yasushi Kurihara, Daisuke Tachibana

**Affiliations:** 1 Obstetrics and Gynecology, Osaka Metropolitan University Graduate School of Medicine, Osaka, JPN

**Keywords:** complete septate uterus, müllerian duct anomaly, pregnancy in both uterine cavities, twin pregnancy, cesarean section

## Abstract

A complete septate uterus, resulting from the abnormal fusion of the Müllerian ducts, is a rare uterine anomaly in which the uterine cavity is divided by a septum, resulting in two separate uterine cavities. In this case, we encountered a patient with a complete septate uterus complicated by a dichorionic diamniotic twin pregnancy, with each fetus developing in separate uterine cavities. During the course of the pregnancy, the placenta of the trailing twin (Twin B) was attached to the septum. As the pregnancy progressed, the septum became progressively thinner, and Twin B exhibited growth restriction.

## Introduction

Uterine anomalies are significant congenital abnormalities in gynecology, with a reported prevalence of 0.5% [1]. Among these, a septate uterus is caused by a failure in the fusion of the Müllerian ducts during embryonic development, resulting in partial or complete division of the uterine cavity by a septum. Normally, this septum disappears during fetal development after the Müllerian ducts fuse, but incomplete resorption leaves the septum intact, thus forming a septate uterus. While the etiology of Müllerian anomalies is considered multifactorial, the underlying causes remain unclear [2].

In the case of a complete septate uterus, the uterine cavity is separated into two separate compartments, and simultaneous pregnancies in both uterine cavities are extremely rare. Only a limited case has been reported in the literature, and there is no established consensus regarding the mode of delivery or cesarean section methods for such cases [3-6].

Here, we report a rare case of a dichorionic diamniotic twin pregnancy in a patient with a complete septate uterus, in which pregnancies occurred in each uterine cavity. During cesarean delivery, we perforated the septum within the uterine cavity to deliver Twin B.

## Case presentation

**Figure 1:**
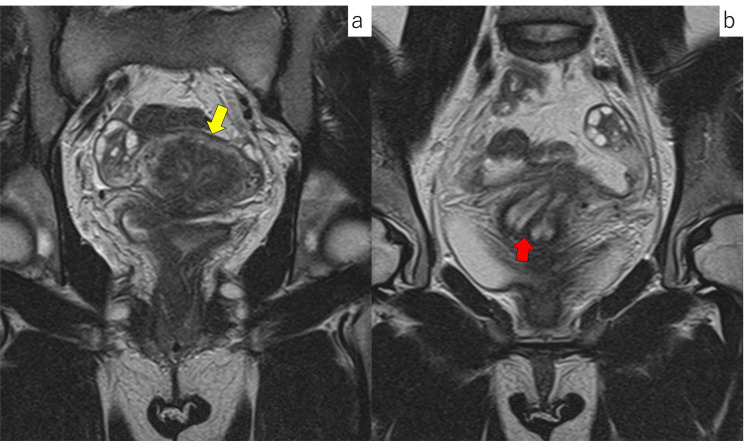
T2-weighted MRI images before pregnancy. (a) The appearance of the uterus revealed no indentation at the uterine fundus (yellow arrow), and the septum was separating the uterine cavity. (b) The septum extended to the cervix (red arrow). MRI: magnetic resonance imaging

**Figure 2:**
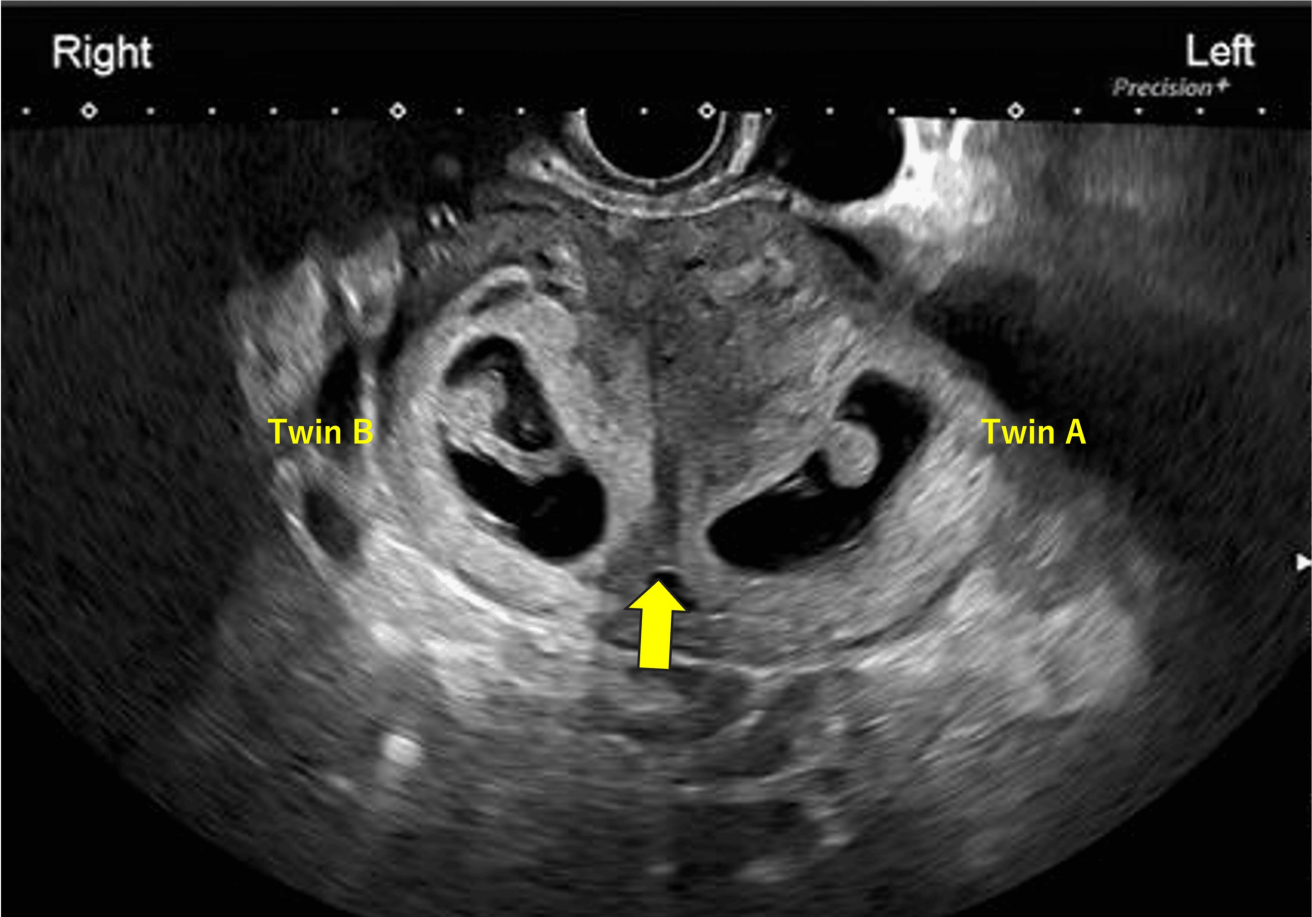
Transvaginal ultrasougraphy image at eight weeks of pregnancy. Gestational sac of Twin A was observed in the left uterine cavity, and Twin B  in the right, separated by the septum (yellow arrow).

A 30-year-old woman (gravida 3, para 1; one spontaneous abortion, one vaginal delivery) underwent a resection of a vaginal septum at the age of 26 years. At that time, magnetic resonance imaging (MRI) confirmed the presence of a septum extending from the uterine fundus to the cervix (Figure 1). Both maternal kidneys were normal on transabdominal ultrasound examination. Apart from the uterine septum and the resected vaginal septum, no other developmental abnormalities were observed. She conceived in the current pregnancy using a timed intercourse method following a five-day course of letrozole at 5 mg/day. At her first visit to our hospital at eight weeks of gestation, transvaginal ultrasonography revealed gestational sacs in both the left and right uterine cavities, separated by the septum (maternal left side: Twin A, maternal right side: Twin B) (Figure 2).

**Figure 3:**
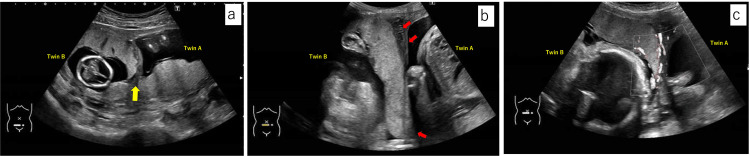
Abdominal ultrasound sonography. (a) Ultrasound image at the gestational age of 14 weeks revealed the placenta of Twin B partially attached to the septum (yellow arrow). (b) Ultrasound image at the gestational age of 28 weeks showed the thinned septum (red arrows). (c) Ultrasound image at the gestational age of 32 weeks showed the flow of the maternal blood floor of Twin B.

**Figure 4:**
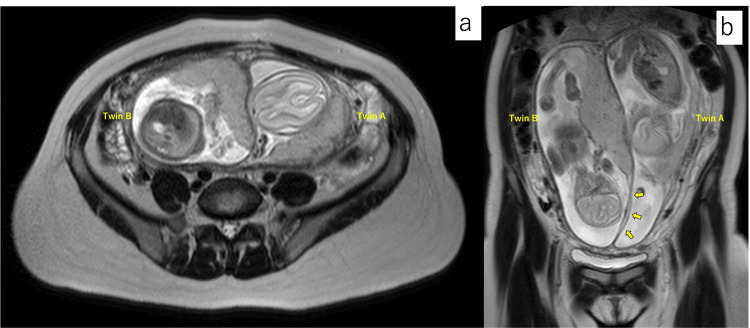
MRI T2-weighted images at 20 weeks of pregnancy. (a) Axial image showed the placenta of Twin B attached to the septum. (b) Coronal image showed the thin septum without placenta of Twin B (yellow arrows). MRI: magnetic resonance imaging

The pregnancy progressed without complications, and at 14 weeks of gestation, an ultrasound revealed that the placenta of Twin B had partially attached to the uterine septum at the anterior wall of the uterus (Figure 3a). As the pregnancy progressed, the septum was observed to have become progressively thinner (Figures 3b, 3c). To evaluate the uterine morphology and the relationship between the placenta and septum, an MRI was performed at 20 weeks of gestation. Consistent with the ultrasound findings, Twin B’s placenta was attached to the uterine fundus and the septum (Figure 4).

Regarding fetal growth, Twin A grew within the normal range of the fetal growth curve, while Twin B showed growth restriction after 32 weeks of gestation. During the pregnancy, no perinatal complications, such as preterm labor or hypertensive disorders with pregnancy, occurred. Due to the breech presentation of Twin A, an elective cesarean section was performed at 37 weeks and 2 days of gestation.

**Figure 5:**
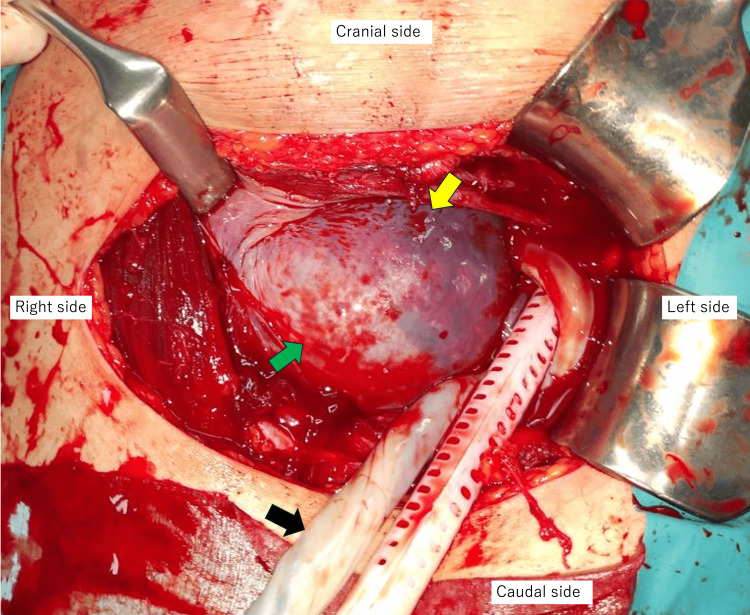
Intraoperative findings after delivery of Twin A. Placenta of Twin B was visible through the septum (yellow arrow), and the septum without the placenta on the cervical side allowed visualization of Twin B’s amniotic cavity through the septum (green arrow). The umbilical cord of Twin A was emerging from the left uterine cavity outside the uterus (black arrow).

A Pfannenstiel transverse incision was made, and the absence of uterine rotation was confirmed under direct visualization by observing the round ligaments on both sides. To deliver Twin A, a transverse incision was made on the left of the lower uterine segment, thus accessing the left uterine cavity, and the baby was delivered in breech presentation. Enlarged blood vessels on the fundal side of the septum were observed, along with the vascular bed of Twin B’s placenta visible through the septum, and the septum without the placenta on the cervical side allowed visualization of Twin B’s amniotic cavity through the septum (Figure 5).

**Figure 6:**
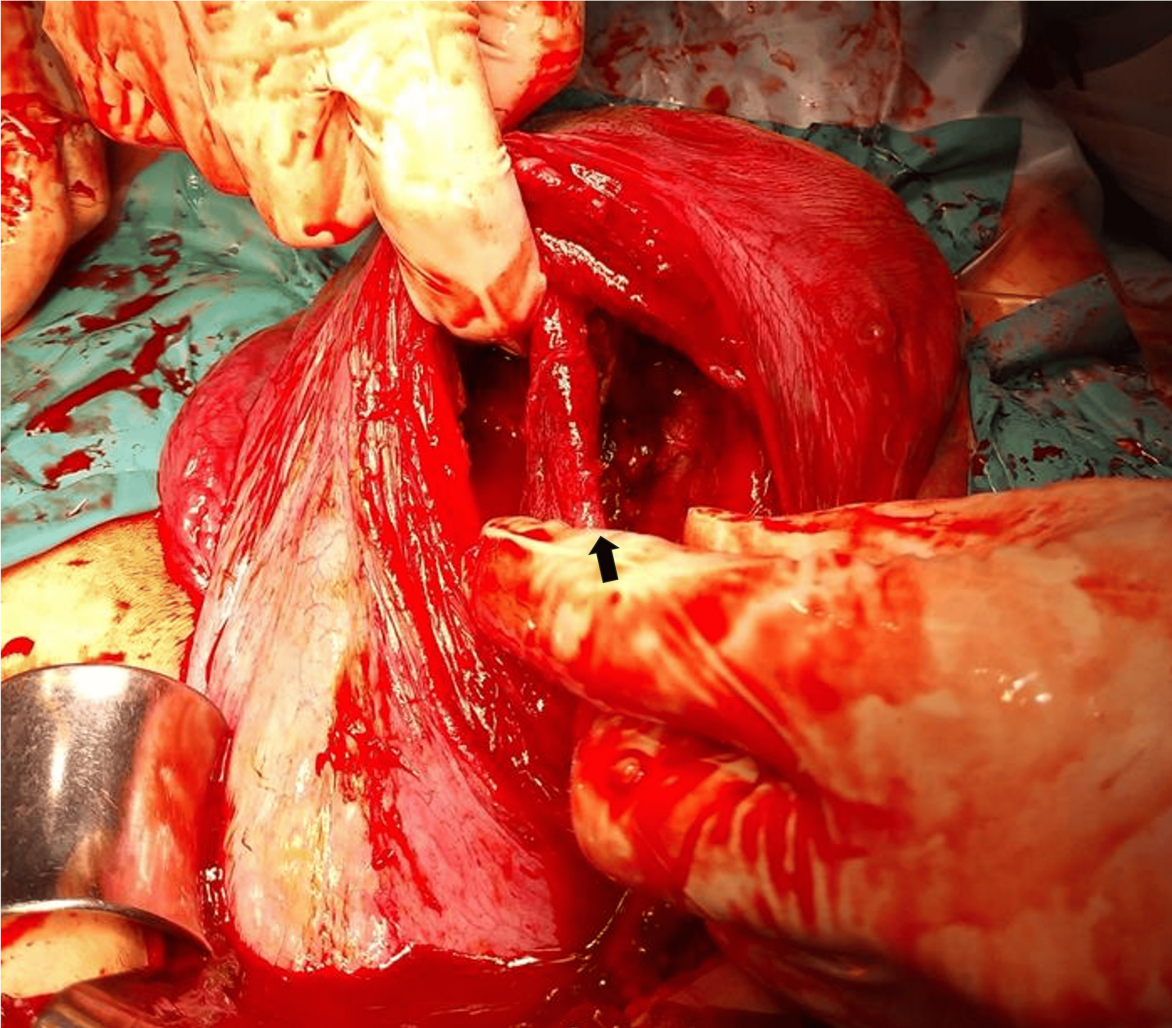
Intraoperative findings of the uterus after removal of both placentas. The image shows the uterine cavities after removal of both placentas. The septum where the placenta of Twin B was attached was relatively thick (black arrow), and the perforated membranous septum was trimmed. There was minimal bleeding from the remaining septum.

**Figure 7:**
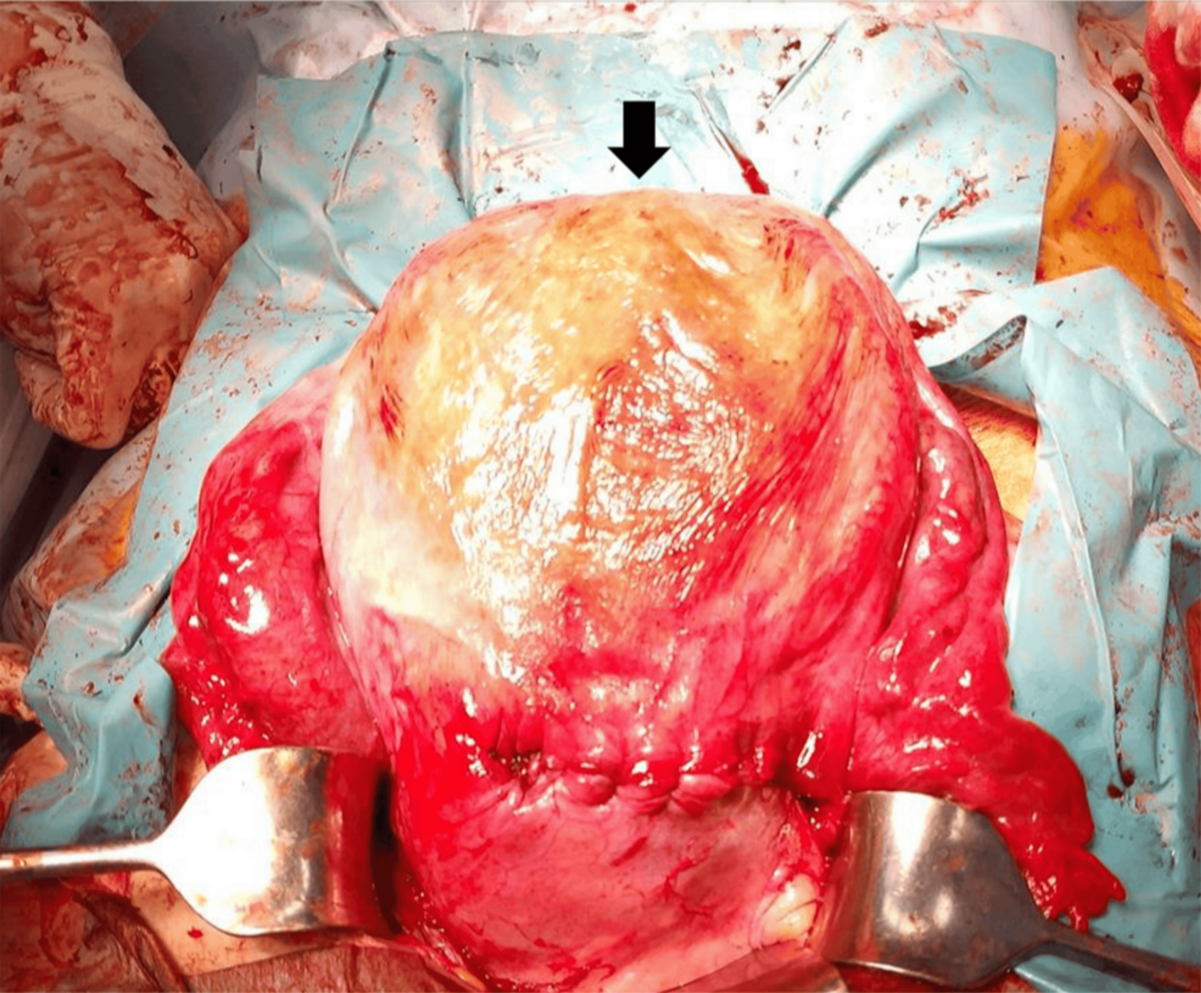
Intraoperative findings of the uterus after uterine suturing. No serosal indentation was observed at the uterine fundus (black arrow), and the external appearance was symmetric and normal.

**Figure 8:**
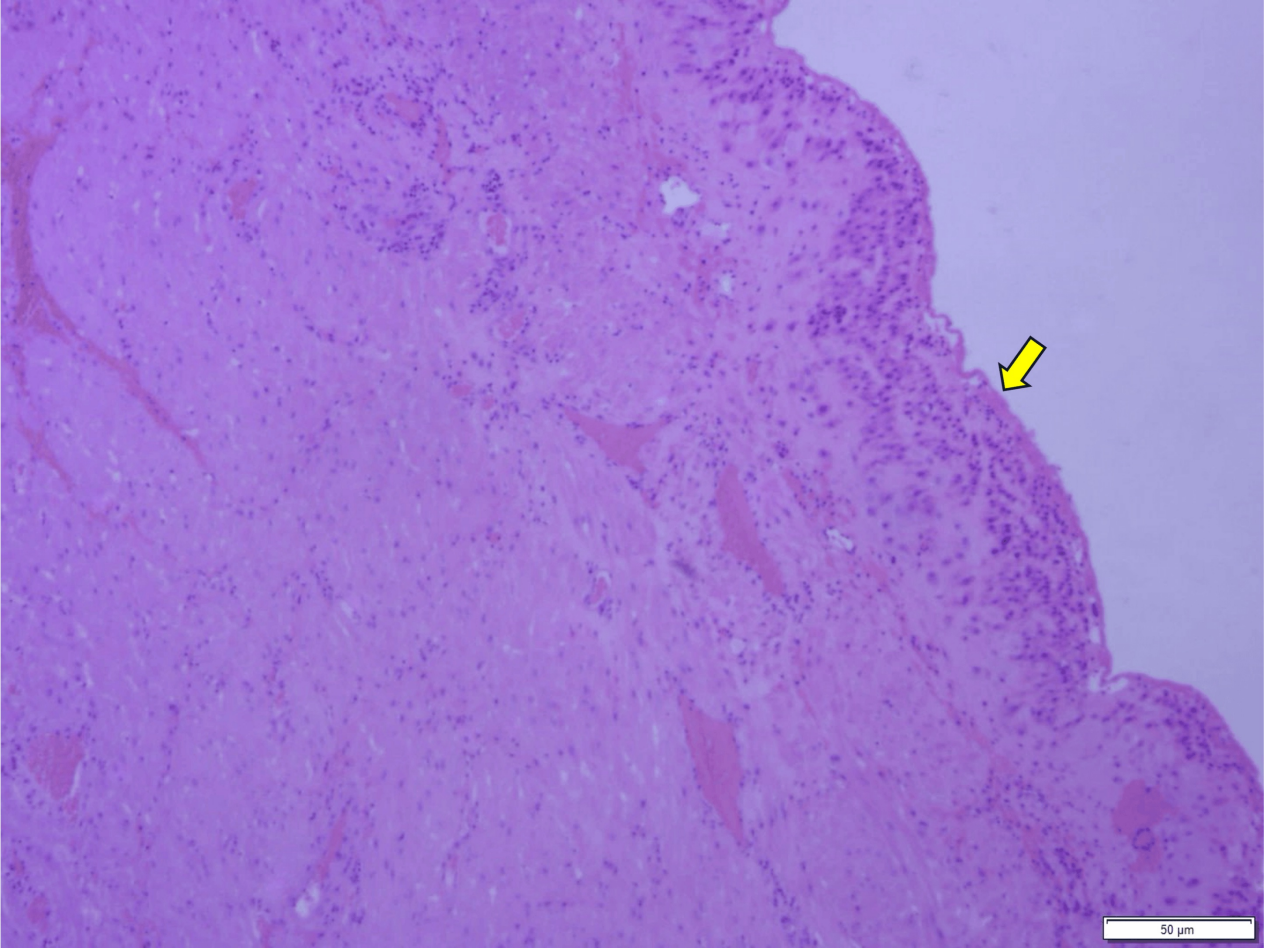
Pathological image of the resected septum. Membranous muscle tissue was observed, and the superficial layer showed decidua (yellow arrow). This is a finding of the normal uterine septum.

To deliver Twin B, we perforated the part of the septum without the placenta, thus accessing the right uterine cavity, and the baby was delivered in cephalic presentation. Both placentas were easily detached and delivered. The perforated septum was membranous, and little bleeding was observed at the perforation site. Given the difficulty of repairing the thin, membranous septum, we excised the site. Observation of the uterine cavity before closure revealed residual septum at the uterine fundus (Figure 6). After suturing the uterus, no serosal indentation was observed at the uterine fundus, and the external appearance was symmetric and normal, thus confirming the diagnosis of a septate uterus (Figure 7). A histopathological examination of the excised membranous septum showed myometrial tissue with decidua, consistent with a uterine septum (Figure 8). The birth weights of the twins were 2,700 g (-0.26 SD) for Twin A and 2,075 g (-2.13 SD) for Twin B.

## Discussion

The classification of Müllerian anomalies has traditionally been based on the American Fertility Society (AFS) classification. However, this classification lacked a specific categorization for vaginal anomalies, requiring additional descriptive documentation of vaginal and cervical findings [7]. In contrast, the classification proposed jointly by the European Society of Human Reproduction and Embryology (ESHRE) and the European Society for Gynaecological Endoscopy (ESGE) in 2013 has been widely accepted in recent years [8]. This classification includes anomalies of the cervix and vagina, thus distinguishing it from the AFS classification. Furthermore, in 2016, the American Society for Reproductive Medicine (ASRM) introduced a modified classification based on the AFS classification, which clarified the differentiation between a septate uterus and bicornuate uterus by assessing the uterine external contour for serosal indentation exceeding 1 cm along the midline [9].

In this case, morphological evaluation via MRI revealed a septum extending from the uterine fundus to the cervix, without serosal indentation exceeding 1 cm on the external contour of the uterine fundus. Based on these findings, the diagnosis was confirmed as a septate uterus rather than a bicornuate uterus. Additionally, the patient had a history of vaginal septum resection before pregnancy, and the presence of duplicated cervices and a non-obstructive vaginal septum led to a diagnosis of ESHRE/ESGE classification U2bC2V1.

A septate uterus arises when the septum between the fused Müllerian ducts fails to be reabsorbed. The completeness of the septum (partial or complete) depends on the timing of the reabsorption defect. The external appearance of the uterus is symmetric and normal. The septum is composed of fibrous or muscular tissue with poor vascularization, which can cause localized uterine contractions and has been associated with adverse obstetric outcomes, including placental abruption, miscarriage, preterm delivery, and fetal growth restriction [10-12].

Venetis et al. reported a meta-analysis that evaluated the reproductive outcomes of congenital uterine anomalies, including septate uterus. The relative risks (RRs) of adverse outcomes, compared to a control group, were reported as follows: preterm delivery (RR = 2.11; 95% confidence interval (CI) = 1.51-2.94), malpresentation (RR = 4.35; 95% CI = 2.52-7.50), fetal growth restriction (RR = 2.54; 95% CI = 1.04-6.23), placental abruption (RR = 4.37; 95% CI = 1.12-17.08), and perinatal mortality (RR = 2.43; 95% CI = 1.10-5.36) [13]. These complications are thought to arise from the anatomical and functional deficits inherent to the septate uterus, particularly its impaired blood flow, which affects normal fetal development. Thus, diagnosing septate uterus before pregnancy is crucial for assessing perinatal risks [14].

There is no standardized delivery strategy for cases of septate uterus, but cesarean delivery rates tend to be higher due to the potential obstruction of fetal passage caused by the septum, especially in twin pregnancies [15]. Lloyd reported the first case of twin pregnancy in separate uterine cavities of a septate uterus in 1927 [3]. Since then, only a few cases have been reported. Clear differentiation between bicornuate and septate uterus became feasible after the ASRM classification in 2016. Reports of twin pregnancies in completely septate uteri, such as the current case, remain exceedingly rare. Apart from this report, a similar case was described by Goulios et al., where a twin pregnancy in separate cavities of a completely septate uterus was achieved naturally after vaginal septum resection [4].

Most previous cases of twin pregnancies in separate cavities of a septate uterus have been delivered via cesarean section, either electively or due to fetal distress or anatomical obstructions, though there are reports of successful vaginal deliveries [5,6].

Osazuwa and Ejenobo described a twin pregnancy in a septate uterus with separate cavities where cervical cerclage was performed prophylactically at 13 weeks to prevent preterm labor and an elective cesarean section was performed at 37 weeks [10]. They initially accessed the right cavity via a classical vertical incision to deliver the first twin, but the extensive septum hindered access to the left cavity. A lower uterine segment incision was added to deliver the second twin. The placenta of the second twin was adherent to the septum, thus necessitating excision of the septum for placental removal.

Goulios et al. reported a case in which the first twin was delivered through a transverse incision in the lower uterine segment, but due to a narrow lower uterine segment on the second twin’s side, a classical vertical incision was made. They avoided damaging the septum to minimize bleeding risks and preserve uterine integrity [4].

In the current case, the cesarean section was initiated with a transverse incision on the lower uterine segment toward the left side to deliver Twin A. After the delivery of Twin A, the thinned septum allowed clear visualization of the amniotic cavity of Twin B. Without adding an additional incision in the lower uterine segment, the septum was perforated to deliver Twin B with minimal difficulty. Bleeding from the perforated septum was negligible, likely due to its poor vascularization. This approach, utilizing the thinned septum for delivery, should be considered an option for cesarean sections in a complete septate uterus. Additionally, no placental adherence was observed in this case, and both placentas were delivered smoothly.

Angiogenesis is critical for fetal and placental development, but the septum region demonstrates impaired angiogenic processes [16]. Reduced expression of vascular endothelial growth factor (VEGF) receptors, including KDR (VEGFR-2) and Flt-1 (VEGFR-1), has been observed in the septum, which may inhibit angiogenesis and limit blood flow to the placenta. This insufficient blood supply, coupled with restricted tissue remodeling in the septum, can lead to fetal growth restriction [16].

In this case, Twin B’s placenta was attached to the septum, likely contributing to the observed growth restriction. However, sufficient placental blood flow allowed Twin B to reach a birth weight of 2,075 g (-2.13 SD), despite being classified as a low-birth-weight infant.

## Conclusions

In this case, we encountered a rare instance of dichorionic diamniotic twin pregnancy within the bilateral uterine cavities of a complete septate uterus. The placenta of Fetus B was attached to the uterine septum and, as previous studies have suggested, impaired angiogenesis in the septum likely contributed to fetal growth restriction in Fetus B. Additionally, due to the breech presentation of Fetus A, a cesarean section was performed. During the delivery of Fetus B, the thinned uterine septum was intentionally perforated to facilitate delivery. The perforated septum had a membranous structure, and no significant bleeding was observed, further indicating the reduced vascularity of the septum. The approach of perforating the septum, as employed in this case, may serve as a viable delivery method for similar cases in the future.
